# Exposed Hematoma After Pacemaker Generator Change

**DOI:** 10.1016/j.jaccas.2021.03.021

**Published:** 2021-07-21

**Authors:** Takahiro Hayashi, Koki Shishido, Kazuki Tobita, Shigeru Saito

**Affiliations:** Department of Cardiology, Shonan Kamakura General Hospital, Kamakura, Japan

**Keywords:** bleeding, DOAC, pacemaker

## Abstract

A pacemaker generator change for a frail woman was performed with a direct oral anticoagulant. After the procedure, a hematoma was exposed. Stopping direct oral anticoagulants in frail patients should be considered before the procedure. (**Level of Difficulty: Beginner.**)

## Central Illustration

**Figure:**
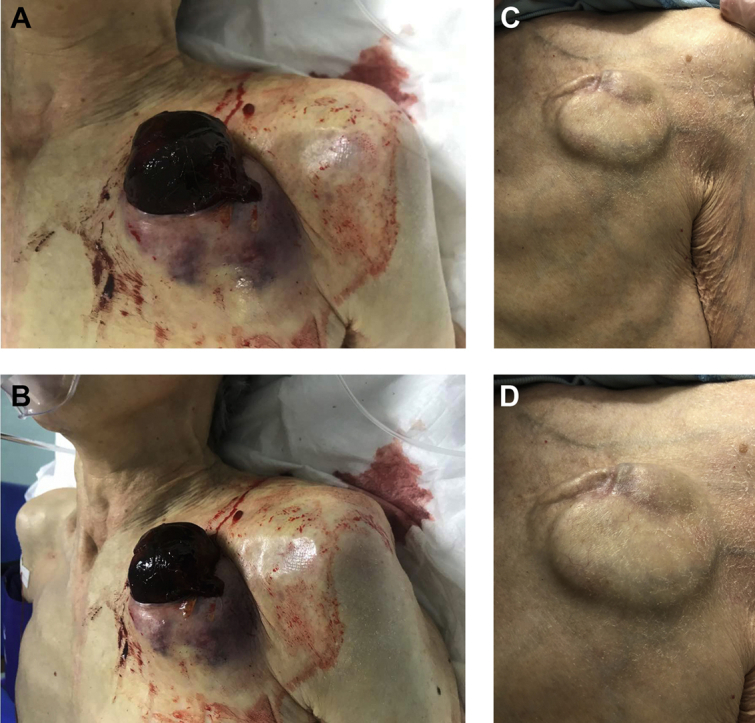

**Figure 1:**
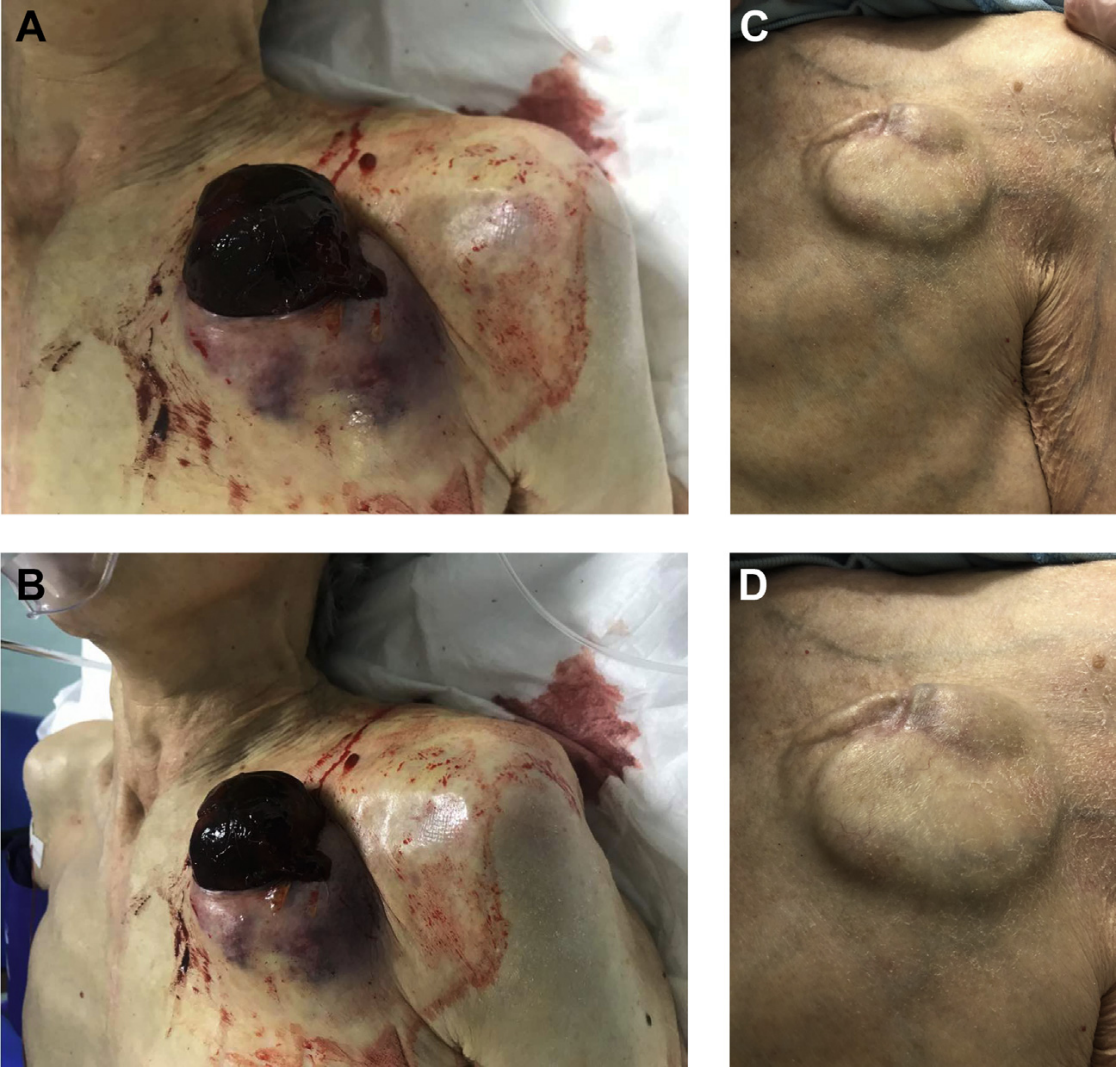
Images After Pacemaker Generator Change and at Follow-Up **(A)** An image of exposed hematoma after pacemaker generator change from the front side. **(B)** An image of exposed hematoma after pacemaker generator change from the lateral side. **(C)** A distant image of the wound at follow-up. **(D)** A close image of the wound at follow-up.

An 83-year-old frail woman (body mass index: 18.7 kg/m^2^) was referred from another hospital for a pacemaker generator change. Her prescriptions included direct oral anticoagulants (DOACs) because of atrial fibrillation. Surgery for a pacemaker generator change was performed without interruption of DOACs. Three days after the operation, a hematoma at the surgical site increased. The surgical site was tightly packed with sterilized gauze to compress the hematoma. After several hours, the hematoma was exposed from the surgical site (Figures 1A and 1B). Re-operation for removing the hematoma was performed. Blood oozing at the surgical site was hemostated with electrical cautery. She was discharged 5 days after re-starting DOACs. At follow-up after surgery, the wound was completely healed, and there were no signs of infection (Figures 1C and 1D).

A recent trial reported that bleeding events after a pacemaker implantation or a pacemaker generator change were not different with or without DOACs (1). Accordingly, discontinuation of DOACs was not recommended in general. However, like our case, stopping DOACs in older adult, Asian, frail, women with small bodies should be considered before the procedure.
